# HERALD (Health Economics using Routine Anonymised Linked Data)

**DOI:** 10.1186/1745-6215-12-S1-A44

**Published:** 2011-12-13

**Authors:** Muhammad J Husain, Sinead Brophy, Steven Macey, Leila M Pinder, Mark D Atkinson, Roxanne Cooksey, Ceri J Phillips, Stefan Siebert

**Affiliations:** 1 College of Medicine, Swansea University, Swansea, SA2 8PP, Wales, UK; 2 College of Human and Health Sciences, Swansea University, Swansea, SA2 8PP, Wales, UK

## Background

Health economic analysis traditionally relies on patient derived questionnaire data, routine datasets, and outcomes data from experimental randomised control trials and other clinical studies, which are generally used as stand-alone datasets. Herein, we outline the potential implications of linking these datasets to give one single joined up data-resource for health economic analysis.

## Method

The Health Information Research Unit (HIRU) at Swansea University has set up the Secure Anonymised Information Linkage (SAIL) databank, which brings together and links a wide range of anonymous patient-level data [1,2]. The linkage of individual level data from questionnaires with routinely-captured health care data allows the entire patient journey to be mapped both retrospectively and prospectively. We illustrate this with examples from a population-based Ankylosing Spondylitis (PAS) cohort [3] by linking patient reported study dataset with the routinely collected general practitioner (GP) data, inpatient (IP) and outpatient (OP) datasets, and Accident and Emergency department data in Wales.

## Potential benefits of data linkage

The linked data system allows: (1) retrospective and prospective tracking of patient pathways through multiple healthcare facilities; (2) validation and clarification of patient-reported recall data, complementing the questionnaire/routine data information; (3) obtaining objective measure of the costs of chronic conditions for a longer time horizon, and during the pre-diagnosis period; (4) assessment of health service usage, referral histories, prescribed drugs and co-morbidities; and (5) profiling and stratification of patients relating to disease manifestation, lifestyles, co-morbidities, and associated costs.

## Results

Using the GP data system we tracked 183 AS patients retrospectively and prospectively from the date of questionnaire completion to gather the following information: (a) number of GP events; (b) presence of a GP ‘drug’ read codes; and (c) the presence of GP ‘diagnostic’ read codes. We tracked 236 and 296 AS patients through the OP and IP data systems respectively to count the number of OP visits; and IP admissions and duration. The results are presented under several patient stratification schemes based on disease severity, functions, age, sex, and the onset of disease symptoms.

## Conclusion

The linked data system offers unique opportunities for enhanced longitudinal health economic analysis not possible through the use of traditional isolated datasets. Additionally, this data linkage provides important information to improve diagnostic and referral pathways, and thus helps maximise clinical efficiency and efficiency in the use of resources.
